# The Cholesterol-Lowering Effect of Oats and Oat Beta Glucan: Modes of Action and Potential Role of Bile Acids and the Microbiome

**DOI:** 10.3389/fnut.2019.00171

**Published:** 2019-11-27

**Authors:** Susan A. Joyce, Alison Kamil, Lisa Fleige, Cormac G. M. Gahan

**Affiliations:** ^1^APC Microbiome Ireland, University College Cork, Cork, Ireland; ^2^School of Biochemistry and Cell Biology, University College Cork, Cork, Ireland; ^3^Quaker Oats Center of Excellence, PepsiCo R&D Nutrition, Barrington, IL, United States; ^4^School of Microbiology, University College Cork, Cork, Ireland; ^5^School of Pharmacy, University College Cork, Cork, Ireland

**Keywords:** microbiome, microbiota, oat beta glucan, bile, propionate, cholesterol

## Abstract

Consumption of sufficient quantities of oat products has been shown to reduce host cholesterol and thereby modulate cardiovascular disease risk. The effects are proposed to be mediated by the gel-forming properties of oat β-glucan which modulates host bile acid and cholesterol metabolism and potentially removes intestinal cholesterol for excretion. However, the gut microbiota has emerged as a major factor regulating cholesterol metabolism in the host. Oat β-glucan has been shown to modulate the gut microbiota, particularly those bacterial species that influence host bile acid metabolism and production of short chain fatty acids, factors which are regulators of host cholesterol homeostasis. Given a significant role for the gut microbiota in cholesterol metabolism it is likely that the effects of oat β-glucan on the host are multifaceted and involve regulation of microbe-host interactions at the gut interface. Here we consider the potential for oat β-glucan to influence microbial populations in the gut with potential consequences for bile acid metabolism, reverse cholesterol transport (RCT), short-chain fatty acid (SCFA) production, bacterial metabolism of cholesterol and microbe-host signaling.

## Introduction

A significant body of evidence demonstrates that consumption of oat products is linked to a reduction in serum LDL cholesterol, a risk factor for the development of cardiovascular disease (CVD) (1–3). Oats are a source of soluble fiber in the form of β-glucan (as well as arabinoxylan, xyloglucan, and other minor components), insoluble fiber, protein, lipids, phenolic compounds, vitamins, and minerals. Whilst other constituents in oats may also have an impact, the cholesterol-lowering activity of oats has been demonstrated to be associated with an increase in viscosity of the gut contents (4) which enhances excretion of bile acids and cholesterol in the feces (5). Indeed, consumption of β-glucan alone can reduce serum cholesterol (6). The weight of evidence in support of a beneficial role of oat β-glucans led the US Food and Drug Administration (FDA) to authorize the use of health claims on oat products attributing lowering of CVD risk to consumption of at least 3 g per day of β-glucan. Cholesterol lowering claims have also been approved in the EU by the European Commission (7–9) and in a number of other jurisdictions including Australia and New Zealand (10), Canada (11), Brazil (12, 13), Malaysia (14), Indonesia (15), and South Korea (16).

We, and others, consider that the cholesterol-lowering properties of oats may not be solely attributable to the viscous properties of β-glucans (17–19). Recent research suggests a significant role for the gut microbiota in the maintenance of cholesterol homeostasis in the host (20). A number of studies have demonstrated the efficacy of probiotics [in particular, probiotic strains with an ability to metabolize host bile acids through bacterial bile salt hydrolase (BSH) activity] in lowering cholesterol in animal models or in humans (21–25). Microbial metabolism of bile acids is known to influence systemic cholesterol metabolism. As cholesterol is a precursor of bile acids, influencing bile acid synthesis provides a means for enhanced excretion of cholesterol thereby lowering serum cholesterol levels in the host (26, 27). Bile acid signaling through the farnesoid X receptor (FXR) and other receptors may also influence host metabolism of cholesterol, for example through the induction of cholesterol transport in the gut (27, 28). Alterations to microbial production of short chain fatty acids (SCFA) including propionate are also likely to have consequences for cholesterol metabolism in the host (29), though the precise mechanisms remain to be elucidated (30). Importantly, oat products and oat β-glucans have been shown to modulate the gut microbiota in human, animal and *in vitro* fermentation systems (19, 31, 32). Therefore, oats (including oat β-glucans) may have a dietary influence upon the host gut microbiota with consequences for bile acid signaling, SCFA signaling, and other effects that are known modulators of host cholesterol homeostasis.

Herein we review the evidence linking components of oats with alterations to host microbiota and discuss potential mechanisms by which such microbiota changes may influence host cholesterol metabolism with a particular focus upon bile acid metabolism. Whilst we appreciate that oat β-glucans may also play a role in post-prandial glucose homeostasis (33) in this review we will predominately focus upon mechanisms by which they lower host cholesterol. Our focus is primarily on the effects of oat β-glucans. However, some reference will be made to the mechanistic effects of barley β-glucans, notably in instances where relevant studies have not yet been performed using oat β-glucans.

## Clinical Evidence for Cholesterol-Lowering Properties of Oats

A large number of individual randomized-controlled trials and subsequent meta-analyses have established a significant effect of consumption of oats or oat β-glucans in reducing LDL cholesterol and improving other markers of cardiovascular disease (CVD) risk (1–3, 34). A meta-analysis of 126 individual studies by Tiwari and Cummins (1) examined the effect of β-glucan intake on measures of blood cholesterol [total cholesterol (TC) and low-density lipoprotein (LDL)-cholesterol] as well as blood glucose levels. The study demonstrated a significant reduction of TC (by 0.6 mmol/L), LDL cholesterol (by 0.66 mmol/L), and TGL/TAG (by 0.04 mmol/L) and an increase in HDL cholesterol (by 0.03 mmol/L) following consumption of oat or barley β-glucan (oat and barley β-glucans are considered bioequivalent with respect to cholesterol-lowering properties). A dose response model demonstrated a decrease in TC with an increase in β-glucan dose but no increased effect in individuals consuming over 3 g/day β-glucan (1). This finding supports FDA recommendations relating to consumption of 3 g/day β-glucan to lower CVD risk (35).

Similarly a meta-analysis of randomized-controlled trials by Whitehead et al. which focused upon consumption of ≥3 g/day oat β-glucan showed a significant reduction in both TC (by 0.30 mmol/L) or LDL cholesterol (by 0.25 mmol/L) (but no effect on HDL cholesterol or TGL) (2). The study found no increased effect in those consuming higher doses of β-glucan, again suggesting that a minimum recommended dose of 3 g/day is sufficient for the cholesterol-lowering effect and is not enhanced through consumption of higher doses.

AbuMweis et al. (36) combined the data from 11 randomized-controlled trials that fitted their weighted criteria based on dose, duration, source of β-glucan, population characteristics and sample size to report that interventions did elicit changes in total and LDL cholesterol levels relative to control subjects, but no dose-response was observed. Reductions in TC of 0.30 mmol/L and reductions in LDL cholesterol of 0.27 mmol/L were reported in response to consumption of ≥3 g/day barley β-glucan.

The lack of a dose-response when consuming levels of β-glucan >3 g/day was noted above. This lack of dose response may reflect variation in the physico-chemical properties of β-glucans used in individual randomized-controlled trials and included in the above meta-analyses. It is known that highly water soluble β-glucan of medium to high average molecular weight (M_w_) is more effective in reducing serum cholesterol than poorly water soluble β-glucan of low M_w_ (37). However, the precise M_w_ of β-glucan can be difficult to establish and may not be accurately reported in randomized-controlled trial data (2). It has also been suggested that the individual food matrix and/or food processing procedures may influence the M_w_ (and therefore bioactivity) of β-glucan and that this is a further confounding factor when comparing data from individual trials (2). The influence of so many variables may suggest that particular meta-analyses are not sufficiently powered to detect a dose-effect when comparing studies which use differing forms of β-glucan with variations in viscosity and bioactivity where such parameters remain unknown (2, 36).

## Bile Acid Synthesis in the Host

Bile acid synthesis and excretion is the main route by which cholesterol is effectively eliminated from the body. In the following sections we provide a basic overview of bile acid metabolism in the host with a particular emphasis upon how the gut microbiota contributes to metabolism of the bile acid pool. These concepts are further expanded in sections Alterations to Gut Microbiota and Effects on Cholesterol Metabolism and Mechanisms by Which Oat β-Glucan may Influence Host Cholesterol Metabolism Through Alterations in BSH Activity of the Microbiome below.

### The Bile Acid Cycle, Cholesterol, and the Role of Microbial BSH

Bile acids are synthesized in liver hepatocytes from cholesterol by cytochrome enzymes (CYPs). Approximately 500 mg of cholesterol is converted to bile acid (BA) on a daily basis (38). Prior to secretion and storage in the gall bladder primary bile acids chenodeoxycholic acid (CDCA) and cholic acid (CA) are conjugated to either a taurine or a glycine molecule to aid their solubility and excretion from the liver. The majority of conjugated bile acids are reabsorbed in the terminal ileum, with 5% excreted in the feces [see (39) for a review]. Conjugated bile acids are released postprandially from the gall bladder into the small intestine and are subject to enzymatic modifications by the bile salt hydrolase (BSH) activity of the microbiota to liberate them from their cognate amino acid. This renders them susceptible to further microbial modification to form secondary bile acids lithocholic acid (LCA) from CDCA and deoxycholic acid (DCA) from CA. This activity is completed by specific members of the colonic microbiota [the Eubacterium and Clostridium XIVa clusters (40)] although gene analyses suggest that other microbial representatives may be capable of carrying out these reactions [reviewed by Long et al. (39)]. Therefore, while the liver dictates bile acid production, the gut microbiota is responsible for the diversity of BAs derived from the bile acid CA and CDCA families and it also influences reuptake or enterohepatic circulation. Alterations to the range and relative profile of bile acids is a reliable readout of microbial changes in the gut and such changes are particularly evident in disease states including metabolic syndrome, inflammatory bowel diseases and Type II diabetes [see (41, 42) for reviews]. Therefore, the dietary effects of oat β-glucan on the microbiota (outlined in section Alterations to Gut Microbiota and Effects on Cholesterol Metabolism), are likely to impact bile acid profiles in the host with potential consequences for metabolism and signaling.

Bile acids are ligands for the farnesoid-X-receptor (FXR) which is a nuclear receptor that is central to energy and metabolic regulation in a range of different tissues (43). Microbially-modified and unconjugated bile acids are the most potent natural FXR ligands with CDCA < LCA< DCA< CA in order of affinity and activation strength, while ursodeoxycholate (UDCA) and murine tauro-β-muricholic acid can hinder FXR activity (27, 44, 45). FXR is widely distributed in tissues including the intestine and the liver. FXR therefore acts as a bile acid sensor in the intestine and as a controller of bile acid synthesis in the liver (46). It controls bile acid synthesis by a variety of mechanisms. Agonism of FXR in the intestine induces production of the endocrine hormone fibroblast-growth-factor 19 [FGF19 (in humans) FGF15 (in mice)] which enters the circulation and activates specific receptors on hepatocytes to reduce bile acid synthesis (through down-regulation of a key enzyme CYP7A1) (47–49). Alternatively a reduction in engagement of the FXR may enhance expression of regulatory networks that are inhibited by FXR such as the liver orphan receptor (LXR) regulon (24). Another layer of cross-talk from the intestine to the liver acts through enterohepatic re-circulation of bile acids. On reaching the liver, circulating bile acids activate FXR directly to ultimately inhibit *CYP7A1* transcription, again reducing bile acid synthesis (44).

The importance of FXR in host cholesterol metabolism is highlighted by studies using FXR knock-out mice or specific chemical agonists of the FXR [reviewed in Li and Chiang (27)]. Knock-out of FXR in mice results in elevated LDL-C (50), whereas stimulation of the FXR in hypercholesterolaemic mice (using bile acids or specific agonists) results in a lowering of HDL-C and LDL-C (51). More recently, the intestinal FXR agonist Fexaramine (Fex) was shown to induce FGF15 and to lead to broadly beneficial metabolic effects including reduced weight gain in mice fed a high fat diet (52) and reduced serum cholesterol in a mouse model of diabetes (53). The precise mechanisms by which FXR contributes to cholesterol metabolism in the host remain unclear but are thought to involve regulation of fatty acid metabolism as well as Reverse Cholesterol transport (RCT) and Trans-intestinal Cholesterol excretion (TICE) (outlined below) (27, 54).

### Reverse Cholesterol Transport (RCT) and Trans-intestinal Cholesterol Excretion (TICE)

In addition to the incorporation of cholesterol into bile acids and subsequent bile acid excretion, other mechanisms contribute to the systemic control of host cholesterol. RCT is a mechanism for directly transporting cholesterol from the tissues to the liver for excretion into bile, and ultimately in feces. RCT relies upon cholesterol loading onto HDL particles which can remove cholesterol from the tissues, notably from macrophage foam cells in the artery wall [reviewed in Temel and Brown (55) and Tall and Yvan-Charvet (56)]. HDL-cholesterol then enters hepatocytes via specific receptors and the cholesterol is secreted directly into bile for excretion via the specific transporters ABCG5/G8. This represents a mechanism by which HDL-cholesterol is thought to be associated with reduced cardiovascular disease risk.

More recent work has revealed a supplemental system for Trans-intestinal cholesterol excretion (TICE) directly into feces through enterocytes in the proximal small intestine. The model proposes that cholesterol is removed from HDL particles in the liver and loaded onto ApoB-containing lipoproteins which migrate to the small intestine where the particles are transported across enterocytes and the cholesterol is excreted into the intestinal lumen. Again cholesterol excretion is via the ABCG5/G8 transport system, in this case expressed in enterocytes (55). Importantly genes that encode essential components of both RCT and TICE are regulated via FXR (27). These include ApoA1 which encodes a component of HDL particles and the ABCG5/G8 transport system (28). This suggests that bile acid signals (and therefore the microbiota) can modulate both RCT and TICE (section Mechanisms by Which Oat β-Glucan May Influence Host Cholesterol Metabolism Through Alterations in BSH Activity of the Microbiome below).

## The Viscous Nature of β-Glucan and Existing Proposed Mechanism for Cholesterol-lowering

The β-glucan polysaccharide forms a viscous liquid suspension in solution, a characteristic which is predicted to occur under physico-chemical conditions encountered in the GI tract. Intestinal viscosity of β-glucan is determined by its concentration, solubility and M_w_, features that may influence variation in clinical effects seen across different controlled trials. Indeed, recent studies have determined the effects of increasing viscosity upon physiological efficacy. In a large randomized controlled trial the capacity of oat products to lower serum cholesterol was directly proportional to the M_w_ of the β-glucan component, with high (2.2 million g/mol) and medium (850,000 g/mol or 530,000 g/mol) M_w_ β-glucans significantly reducing LDL cholesterol and a low (210,000 g/mol) M_w_ β-glucan proving ineffective (4). Furthermore both increasing viscosity (57) or increasing M_w_ (58) of β-glucan have been shown to increase the ability to regulate post-prandial glucose concentrations in human subjects. The beneficial effects of high M_w_ oat β-glucan are therefore thought to be related to an ability to form a viscous solution in the intestine.

The mechanisms by which viscous β-glucans modulate host cholesterol are thought to be linked to modulation of host bile acid metabolism (59). Viscous β-glucan is hypothesized to interact with bile acids and prevent their re-adsorption in the terminal ileum. This results in increased fecal excretion of bile acids thereby increasing the requirement for *de novo* synthesis of bile acids from cholesterol, a mechanism which lowers systemic LDL cholesterol (59). In support of this, a number of animal studies (60, 61) and human intervention studies have shown elevated fecal bile acid excretion following consumption of oats or β-glucan (5, 62–64). This is matched by evidence for elevated *de novo* bile acid synthesis following consumption of oats both in animals (through measurement of activity of relevant liver enzymes including Cyp7A1) (61) or through measurement of 7 alpha-hydroxy-4-cholesten-3-one (HC) in humans (a marker for bile acid synthesis) (61, 64). A comprehensive study in pigs demonstrated that oat β-glucan feeding increased bile acid excretion during the early feeding period but that bile acid excretion actually decreased in this group following dietary adaptation. The study pointed to alterations in gut physiology, reduced bile acid uptake, and a reduction in cholesterol absorption along with possible microbiota changes that could explain the reduction in systemic cholesterol levels in the β-glucan-fed group (65). The authors indicated that oat β-glucan significantly influenced bile acid and cholesterol metabolism in the host along with a likely beneficial (prebiotic) effect on the gut microbiota which enhanced both the generation of the secondary bile acid UDCA and cholesterol digestion in the gut (65). The possible effects of such microbiota-mediated mechanisms are outlined further in the sections below.

## Alternative Mechanisms by Which Oat β-Glucan May Affect Host Cholesterol Metabolism

### Alterations to Gut Microbiota and Effects on Cholesterol Metabolism

Whilst the precise mechanisms remain to be elucidated it is clear that the gut microbiota plays a significant role in host cholesterol homeostasis. Very early studies indicate that antibiotic treatment of mice inhibits cholesterol metabolism leading to accumulation of systemic cholesterol (66). Also, germ-free rats accumulate greater levels of cholesterol from elevated cholesterol diets compared to conventionally raised animals (67). Germ free rats demonstrated lower levels of systemic catabolism of dietary cholesterol (68) and also showed reduced fecal excretion of both total sterols and bile acids in particular (69). The data suggest that increased bile acid synthesis from cholesterol is a mechanism for lowering of systemic cholesterol levels (68) and is influenced by the activities of the gut microbiota. Furthermore, there is significant evidence that transient alteration of the microbiota through the administration of probiotic bacteria can be beneficial in lowering of systemic cholesterol (see sections below). The data suggest a role for the microbiota in the maintenance of cholesterol homeostasis in the host and suggest that alteration of the community structure of this microbial population has the capacity to influence cholesterol metabolism (70). More detailed studies are necessary in order to pinpoint specific microbial genera or species in the gut which may influence host cholesterol metabolism. Such information is emerging for models of lipid metabolism, weight gain and adiposity. For instance, murine studies pointed to alterations in the relative ratios of two major phyla, Bacteroidetes, and Firmicutes in promoting weight gain (71, 72). The findings also correlated with human studies in obese volunteers subjected to a calorie-restricted diet (73) and the obesity phenotype was transferable by transplant of the microbiota from either obese mice or obese humans to microbiota naïve mice, thereby showing a functional role of the microbiota in this phenomenon (74–76). Other studies have shown a clear link between microbial gene richness and metabolic health. Individuals with low gene richness in the microbiota are more likely to display increased adiposity and dyslipidemia (77).

β-glucan is resistant to depolymerization by gastric and pancreatic enzymes and transits to the colon for microbial fermentation. Alteration of bile acid metabolism is also known to impact the microbiota (78) and is therefore a further possible mechanism by which β-glucans may modulate microbial gut populations.

There is significant evidence from models of increasing complexity (from *in vitro* fermentation models, to rodent to porcine models) and human intervention studies that oat fibers have a significant impact upon the compositional structure of the gut microbial community. For critical reviews of the effects of β-glucan upon the microbiota the reader is referred to Jayachandran et al. (79) and Sanders et al. (80). Relatively simple *in vitro* fermentation studies which mimic the human colon using human fecal bacterial populations allow for highly controlled analyses of bacterial responses to dietary components but lack the biological complexity of *in vivo* models. *In vitro* fermentation studies have shown that addition of oat or barley β-glucan directly promotes the growth of gut bacterial populations (including the *Clostridium histolyticum* subgroup and Bacteroidetes/Prevotella groups) (81). A recent study using an *in vitro* batch culture system demonstrated that oat β-glucan induced proliferation of Bacteroidetes but was not Bifidogenic. In contrast growth of Bifidobacteria was stimulated by oat-derived polyphenols (82). In another study oat flakes promoted growth of the Bacteroides/Prevotella group or *Bifidobacterium* group in a fecal slurry with effects related to the size of the oat flakes (31). A recent study indicates the ability of oat β-glucan to promote growth of *Prevotella* and *Roseburia* species in an *in vitro* fermentation with concomitant production of the short chain fatty acids (SCFA) propionate and butyrate (83). Overall these studies indicate that oat β-glucan and other components of whole oats can influence populations of biologically relevant bacterial taxa.

Recent studies in mice demonstrated that oat β-glucan feeding increases populations of *Bacteroides* species and *Prevotella* species whereas bacteria from the phylum Firmicutes were decreased (84). Zhou et al. similarly showed that whole grain oat flour causes significant alterations to microbiota community structure relative to a control diet with alterations to Prevotellaceae, Lactobacillaceae, and Alcaligenaceae families (85). Importantly the microbiota changes correlated with a significant lowering of total cholesterol and non-HDL cholesterol in animals fed whole grain oat flour (85). Ryan et al. showed a significant reduction in markers of cardiovascular disease risk in an *apoE* deficient mouse model following feeding with oat β-glucans which correlated with an increase in the population of the family *Verrucomicrobia* and elevated production of n-butyrate (32). This is particularly interesting as *Akkermansia muciniphila* (a key member of the *Verrucomicrobia*) has been functionally linked to improved gut barrier function, reduction in obesity, and improved metabolic health (86). Early studies in rats utilized a culture-based approach and demonstrated that feeding of oat flour formulations led to an increase in Bifidobacteria populations in the gut (87). Insoluble high viscosity oat β-glucan enriched for Clostridium cluster I in a pig model with associated increases in butyrate production (88).

When microbial composition studies were performed in humans, low M_w_ barley β-glucan did not appear to alter microbial representation however high M_w_ barley β-glucan was associated with higher levels of the phylum Bacteroidetes while Firmicutes levels were reduced (89). These alterations were accompanied by reductions in CVD risk factors including BMI, blood pressure and circulating triacylglycerol (TAG) over the 35-day study period and the authors identified specific microbial taxa whose abundance correlated with markers of disease risk (including total cholesterol and LDL-C) (89). Another study used fluorescence *in situ* hybridization with probes specific for selected bacterial genera and showed that consumption of an oat-based granola breakfast cereal was associated with a cholesterol-lowering effect concomitant with elevated *Bifidobacterium* and *Lactobacillus* species. As these species are associated with BSH activity and as members of these species have been previously used as probiotics which can lower serum cholesterol levels, the authors suggested that the lowering of serum cholesterol in this study may be linked to alterations to bile acid metabolism and that further studies are warranted. No significant changes were seen in particular species of *Bacteroides, Atopobium*, or *Clostridium* targeted in this study (19).

Overall, intervention studies utilizing sources of β-glucan suggest that consumption can promote alterations to the gut microbiota with some studies suggesting a potentially beneficial (prebiotic) effect (19).

### Mechanisms by Which Oat β-Glucan May Influence Host Cholesterol Metabolism Through Alterations in BSH Activity of the Microbiome

Evidence from *in vitro* fecal fermentation studies and rodent and human intervention studies suggest that oat β-glucan consumption increases levels of bacteria in the gut with known BSH activity (reviewed above).

A variety of studies have demonstrated an elevation of *Bifidobacterium, Bacteroides*, and *Lactobacillus* species in the gut following oat β-glucan consumption. These bacterial genera are known to predominately contain BSH-positive species (90). There is therefore good evidence for an effect of oat β-glucan on the host microbiota with a predicted influence upon those species that are BSH positive. This would suggest that consumption of oat β-glucan has the capacity to alter host bile acid profiles. However, further work is necessary to determine if oat β-glucan can effectively modulate host bile acid profiles in humans as predicted by these microbiota analyses.

There is good evidence that BSH-active probiotic interventions can reduce serum LDL-C providing a direct link between elevated BSH activity and regulation of host cholesterol [reviewed in Jones et al. (24) and below]. Whilst the evidence for a cholesterol-lowering activity of BSH is strong the precise mechanisms remain elusive and most likely reflect an alteration in both the physico-chemical properties of bile acids and the molecular signaling potential of the bile acid pool for FXR.

Bacterial BSH activity is known to alter the host bile acid signature through deconjugation of conjugated bile acids. Unconjugated bile acids have reduced micellular activity and therefore are less effective mediators of cholesterol absorption in the host relative to conjugated bile acids (24).

In support of this, administration of a strongly BSH positive probiotic *L. reuteri* NCIMB 30242 strain to humans lowered serum LDL-C and lowered absolute plasma concentrations of plant sterols (surrogate markers of cholesterol) suggesting decreased inward transport of cholesterol in the gut (23). Therefore, elevated bacterial BSH activity is likely to directly reduce cholesterol uptake from the lumen and this may provide a general mechanism by which BSH regulates systemic cholesterol in the host (24). Unconjugated bile acids are also more likely to be eliminated in the feces thereby driving a requirement for *de novo* bile acid synthesis and an associated reduction of systemic cholesterol (68). Indeed, Joyce et al. (25) showed that expression of highly active *L. salivarius* BSH could significantly reduce LDL cholesterol, total cholesterol and also serum triglycerides in mice. In humans BSH-active *L. acidophilus* administered over 6 weeks could reduce plasma levels of both total cholesterol and LDL-cholesterol (91). BSH active *L. reuteri* NCIMB 30242 significantly reduced LDL-C, total cholesterol in a human randomized controlled study with elevated free bile acid levels detected in circulation (22, 23).

The reduced reabsorption of bile acids also reduces their potential to interact with FXR and may lead to a reduction in stimulation of FXR. However, unconjugated bile acids also can act as potent ligands for the FXR and are also the substrates for further bacterial conversions of bile acids to secondary bile acids which are also potent FXR agonists. Therefore, another possible hypothesis is that FXR is activated in the gut by BSH activity leading to increased FXR signaling in the gut and expression of the hormone FGF19 by enterocytes leading to a reduction in hepatic bile acid synthesis. More research is necessary to understand the chemical and physiological parameters which dictate whether FXR is stimulated through local bacterial BSH activity. In the absence of such research we herein consider the evidence for two potential mechanisms by which elevated BSH activity may impact host systemic cholesterol levels. In hypothesis 1 FXR is not activated and *de novo* bile acid synthesis is increased. In hypothesis 2 FXR is activated and FGF19 is elevated resulting in a reduction in bile acid synthesis and an increase in other mechanisms by which cholesterol levels are potentially modulated in the host. Given a number of recent studies which show a decrease in FXR activation in the gut following administration of BSH-active probiotics we favor hypothesis 1 as most likely to represent a scenario in which BSH activity is increased in the gut microbiota following dietary interventions (as is potentially the case for β-glucan consumption).

#### Hypothesis 1: Elevated Bacterial BSH Activity Can Reduce Engagement of FXR in the Intestine, Increase Bile Acid Excretion and Increase de novo Synthesis of Bile Acids in the Liver (Figure 1)

Conjugated bile acids are actively reabsorbed via specific transport systems into enterocytes in the ileum whereas unconjugated bile acids are not subject to this specific reuptake system and are passively absorbed at a lower rate (92). BSH activity decreases the levels of conjugated bile acids which can be actively transported and recent *in vivo* evidence suggests that the resulting deconjugated bile acids are less efficiently reabsorbed into enterocytes (93). Unconjugated bile acids then enter the colon where conversions to secondary bile acids can take place (94) or are excreted in feces. As the FXR is an intracellular nuclear receptor lower levels of cellular adsorption of bile acids will lead to a lowering of FXR activation in the terminal ileum.

**Figure 1:**
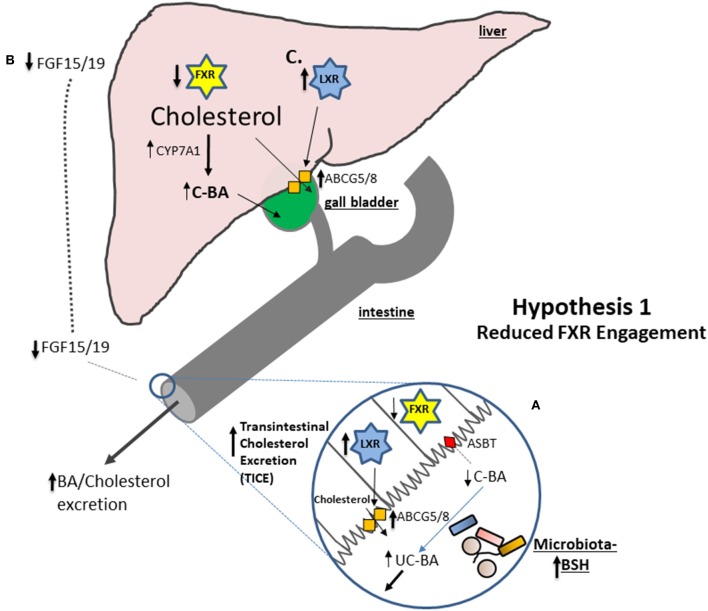
A number of studies suggest that an increase in microbial BSH activity results in a reduction in stimulation of intestinal FXR. **(A)** Conjugated bile acids (C-BA) are rapidly absorbed in the terminal ileum but BSH activity reduces the amounts of C-BA locally leading to decreased entry of bile acids (BA) into the cell and reduced engagement of Farnesoid X receptor (FXR). Reduced FXR potentiates the capacity for LXR to become activated and studies show that liver orphan receptor (LXR) activation stimulates the Transintestinal Cholesterol Excretion (TICE) pathway in the gut leading to net cholesterol excretion. **(B)** Reduced engagement of FXR in the ileum reduces fibroblast growth factor 15 (in mice) or 19 (in humans) (FGF15/19) preventing feedback inhibition of *Cyp7A1* and increasing *de novo* synthesis of bile acids from cholesterol thereby reducing systemic cholesterol levels. **(C)** Increased excretion of bile acids reduces hepatic signaling to FXR which results in increased potential for activation of LXR and elevated expression of *Abcg5/8* allowing for excretion of cholesterol into the bile.

In support of this hypothesis it has been shown that monocolonization of germ-free rats with BSH-active bacterial species significantly promotes fecal excretion of bile acids (95). More recently, oral inoculation of rodent models with BSH-producing probiotic bacteria was shown to reduce intestinal FXR activation relative to controls in tandem with an alteration of local bile acid profiles. Inoculation of mice with a highly BSH-active polybiotic mixture of organisms (VSL#3) significantly reduced FGF15 (a marker of FXR activation) and increased expression of hepatic *Cyp7a1* and *Cyp8b1* and increased bile acid synthesis (93) (see Figure 1). In a separate study oral administration of *L. plantarum* CCFM8661 to mice induced similar effects; inhibition of the FXR-FGF axis, elevated *Cyp7a1* expression and elevated bile acid synthesis (96). In another study in mice administered a high fat diet oral inoculation with BSH-active *Lb. rhamnosus* LGG reduced serum cholesterol in concert with a downregulation in FXR transcription in the liver and increased expression of hepatic *Cyp7a1* (but not *Cyp8b1*) (97). An earlier study also linked the cholesterol-lowering effect of a *Lb. plantarum* probiotic strain to an increase in expression of *Cyp7a1* in mice, indicative of downregulation of FXR-mediated feedback (98). Furthermore, a BSH-active probiotic *Lb. reuteri* NCIMB 30242 has been shown to reduce serum LDL-C in humans with a concomitant increase in total bile acid levels (99). As bile acids are synthesized from cholesterol, increased *de novo* synthesis of bile acids contributes to cholesterol catabolism in the host leading to a lowering of systemic cholesterol levels (68).

Another nuclear receptor, LXR, is indirectly repressed by the FXR. Therefore, another consequence of reduced FXR signaling is an elevation of LXR activity. This stimulation of LXR leads to an increase in expression of the cholesterol efflux system ABCG5/8 in enterocytes (100) and increased excretion of cholesterol (54, 101). A recent study demonstrated that the BSH-active probiotic strain *Lactobacillus plantarum* LRCC 5273 reduces serum cholesterol in mice along with an increase in expression of *Cyp7A1*, an increase in both hepatic and gut LXR activity and elevated expression of gastrointestinal ABCG5/8 allied with a decrease in expression of the gene encoding NPC1L1 (a cholesterol uptake system) (102). The authors propose a model in which elevated BSH activity promotes TICE mediated through LXR activation which involves elevated excretion of cholesterol from the system and reduced cellular uptake (102). In support of this another study in mice demonstrated that an increase in BSH activity in the lumen can increase transcription of *Abcg5/8* concomitant with a reduction in serum cholesterol (25).

Whilst bile acid synthesis is controlled by the gastrointestinal-hepatic FXR-FGF axis another mechanism of feedback inhibition is mediated directly via circulating bile acids. Bile acids entering the enterohepatic circulation have the potential to directly influence FXR signaling in the liver, a process which influences *de novo* bile acid synthesis and cholesterol metabolism (103). A consequence of the elevated excretion of bile acids in feces as outlined above may be reduced re-circulation of bile acids and downregulation of liver FXR activity (24). Downregulation of FXR leads to reduction in SHP and consequently elevated activity of LXR (24). Indeed the recent study cited above suggests that elevated BSH activity in the gut results in elevated LXR expression in the liver in mice (102). The consequences of reduced hepatic FXR signaling include increased expression of Cyp7A1 and therefore increased *de novo* bile acid synthesis. In addition, LXR activation leads to the expression of hepatic ABCG5/8 that promotes cholesterol excretion into bile (104) and plays a significant role in the regulation of systemic cholesterol levels (105).

#### Hypothesis 2: Gastrointestinal FXR Is Activated Through Increased Generation of Unconjugated Bile Acids by Microbial BSH Activity, FGF19 Is Elevated Leading to Downregulation of Hepatic Bile Acid Synthesis and Upregulation of RCT and TICE (Figure 2)

Whilst both conjugated and unconjugated bile acids can activate the FXR, unconjugated bile acids generated through elevated bacterial BSH activity have a greater ability to enter target cells without a specific transport mechanism (106). There is evidence that FXR is activated via BSH activity in the gut. One study indicates that administration of a BSH-active probiotic, *Lb. reuteri* NCIMB 30242 to humans lowers LDL-C with levels of FGF19 trending toward an increase (though the increase was not statistically significant). Furthermore, somewhat counterintuitively, there was evidence for an increase in bile acid synthesis in subjects receiving the probiotic so it is difficult to determine whether the FXR-FGF axis was indeed engaged in this study (99). This is in contrast to numerous studies in animals indicating the opposite effect (see mechanism 1). Therefore, further human intervention studies are needed to determine whether FXR may be stimulated by microbial BSH activity in humans. Other studies demonstrate that a reduction in BSH activity through administration of antibiotics (107) or the antioxidant Tempol (108) decreases gut bacterial BSH activity leading to a reduction in gastrointestinal FXR signaling in mice. The corollary of these findings would suggest that a physiological role of microbial BSH is to enhance FXR signaling in the gut (106, 108). However, the effects of Tempol or antibiotics upon the microbiota are so profound in these experiments, that it is difficult to equate these results to those expected following consumption of oat β-glucan where subtle increases in BSH activity akin to probiotic treatments, are expected.

**Figure 2:**
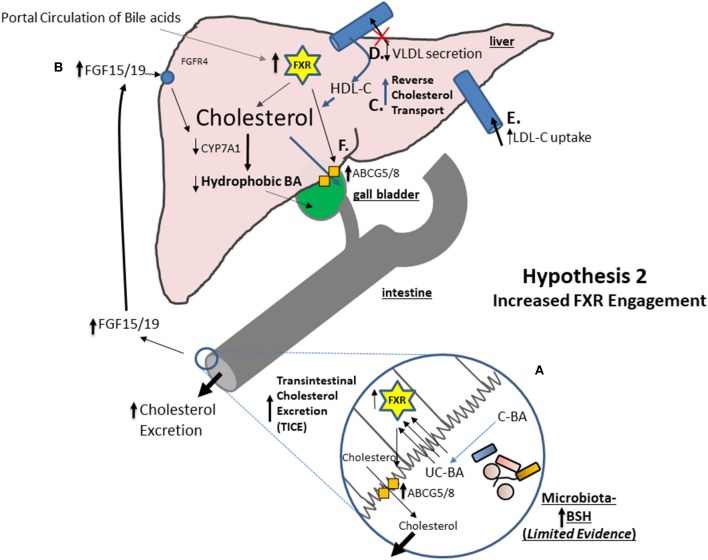
Under specific circumstances which remain unclear the Farnesoid X receptor (FXR) may be stimulated in the gastrointestinal tract. There is some evidence from a single human probiotic study that FXR may be stimulated through BSH activity but further studies are warranted. **(A)** In a model where gastrointestinal FXR is stimulated unconjugated bile acids (UC-BA) may access the FXR through non-specific passage through cell membranes. **(B)** There is good evidence that intestinal FXR activation promotes the Transintestinal Cholesterol Excretion (TICE) system for the net efflux of cholesterol into the feces. FXR activation leads to elevated intestinal production of FGF15/19 which feeds back to inhibit bile acid synthesis. Via a process that involves FXR this results in a net reduction in hydrophobic BA species but a relative increase in hydrophilic BA species which are released into the small intestine. As hydrophilic BAs poorly associate with cholesterol this may reduce cellular uptake of cholesterol in the gut. **(C)** There is also good evidence that elevated hepatic FXR activation increases systemic reverse cholesterol transport (RCT) for the mobilization of cholesterol from macrophages (as HDL-C) to the liver for excretion. **(D)** There is evidence to suggest that engagement of the FXR reduces the secretion of VLDL into the circulation thereby reducing the systemic circulation of this atherogenic molecule. **(E)** There is also evidence to suggest that engagement of the FXR increases the uptake of LDL into the liver from the circulation thereby reducing the systemic circulation of this atherogenic molecule. **(F)** Finally, there is evidence to suggest that FXR activation increases hepatic ABCG5/8 with potential to promote biliary secretion of cholesterol.

There is significant evidence to support a role for FXR signaling in cholesterol homeostasis. However, most of this evidence has been generated through the use of potent FXR-agonists or through experiments using knock-out mice. There is relatively little evidence directly linking these effects to microbiota changes. Oral delivery of FXR agonists has been shown to decrease systemic LDL-C or non-HDL-C (68, 69) and to decrease atherosclerotic plaque formation in mouse models of atherosclerosis (66, 68, 69). Furthermore, mice deleted in FXR display hypercholesterolemia (23, 50, 109). The mechanisms by which the FXR regulates systemic cholesterol metabolism are thought to include regulation of cellular LDL-C uptake, reduction in plasma VLDL, modulation of plasma HDL-C levels, regulation of reverse cholesterol transport (RCT) and possible regulation of trans-intestinal cholesterol excretion (TICE) [reviewed in Li and Chiang (27)]. Studies in animal models suggest that FXR agonists can lower plasma VLDL levels (110, 111). Recent studies demonstrate that FXR agonists reduce hepatic secretion of VLDL by suppressing expression of PLA2G12B, a protein involved in assembly and secretion of potentially atherogenic VLDL (111). In addition, FXR knockout mice demonstrate reduced clearance of plasma HDL-C and further studies suggest that hepatic FXR activation increases RCT from macrophages and fecal excretion of cholesterol (69, 100).

The cholesterol transport system ABCG5/8 is positively regulated by FXR (in addition to regulation via LXR) and there is evidence to suggest that FXR activation increases hepatic ABCG5/8 with potential to promote biliary secretion of cholesterol [reviewed in Li and Chiang (27)]. ABCG5/8 is also expressed in enterocytes where it plays a significant role in the elimination of cholesterol into the gut lumen through TICE [reviewed in de Boer et al. (54)]. In mice engagement of hepatic FXR using an agonist significantly reduced serum cholesterol, increased RCT and increased the hydrophilic bile acid pool. As hydrophilic bile acids less efficiently associate with cholesterol this was thought to be a factor which reduced intestinal absorption of cholesterol thereby lowering systemic cholesterol levels (112). Other studies have indicated that FXR agonists stimulate TICE to significantly increase cholesterol excretion. TICE was not evident in knock-out mice lacking intestinal FXR indicating that this pathway is highly dependent upon FXR activation in the gut (101). However it should be appreciated that activation of LXR in the gut (allied to downregulation of FXR) can also induce the TICE system (see mechanism 1) (101).

### Propionate and Other Short-Chain Fatty Acids (SCFA)

SCFAs are microbial metabolites that are particularly associated with fermentation of dietary fibers. Exposure of fecal bacteria to oat bran fractions using *in vitro* model systems has demonstrated an ability of oat β-glucan to stimulate SCFA production by the gut microbiota (81, 113–115). In many of the studies propionate predominated amongst the SCFAs stimulated by oat bran fermentation (81, 113, 114). Animal studies support the finding that oat fermentation alters the microbiota and elevates SCFA production in the colon. In mice, oat-derived β-glucan consumption led to an alteration to the fecal microbiota and an elevated level of propionate in the colon (84) whilst in another recent study in ApoE^−^ mice feeding of oat β-glucan led to elevated n-butyrate levels (32). In rats, oat β-glucan feeding also increased overall SCFA levels (87, 116, 117). Similarly porcine feeding studies indicate an increase in overall SCFA levels following consumption of oat β-glucans or similar feed additives (118, 119) with elevated butyrate in particular being evident in some studies (120, 121). In one study SCFAs were lower in pigs fed oat products relative to the control (122). However, overall the animal feeding studies suggest an influence of oat β-glucan on the gut microbiota which results in an elevated production of SCFAs.

Whilst studies have examined the effects of oats on human-derived microbial populations in *ex vivo* models, relatively few studies have examined the effects of oat consumption on SCFA production in human intervention studies. In a randomized clinical trial oat β-glucan resulted in reduced cholesterol concomitant with an increase in total SCFA and in particular butyrate (123). A similar study determined that total SCFAs were elevated in subjects fed a β-glucan rich oat bran for 8 weeks (124). Another randomized clinical trial demonstrated the efficacy of bran β-glucans in lowering cholesterol with effects linked to an increase in SCFAs (in particular propionate) concomitant with changes to the microbiota (125). Another study demonstrated an increase in fecal SCFAs in subjects consuming a high M_w_ barley β-glucan in concert with an increase in fecal bile acid excretion (126). The same effects were not seen in subjects consuming a low M_w_ barley β-glucan (126). In contrast, a recent study which investigated the effects of a whole grain oat granola upon microbiota markers failed to show an influence upon fecal SCFA levels despite a significant lowering of TC and LDL cholesterol levels (19). The authors suggested that in future studies measurement of circulating SCFAs would be more informative in order to determine physiologically relevant systemic effects.

Indeed, as SCFAs are rapidly absorbed by enterocytes in the gut their presence may be a rather transient marker of gut microbial activity. In this respect fecal fermentation studies with controlled human microbiota samples may represent an accurate measure of the influence of biotic factors on SCFA production in the gut (as the SCFA will not be absorbed in this model). A study by Carlson et al. recently demonstrated that a commercially available source of oat β-glucan significantly increased propionate production by the microbiota in a human fecal fermentation system (127). The work confirms other earlier studies which demonstrated that addition of sources of oat β-glucan to *in vitro* microbial fermentation systems can increase SCFA (in particular propionate) production (81, 114, 128).

The signaling and health-promoting effects of SCFAs are relatively well-established (129). Luminal propionate engages specific receptors (GPR41 and GPR43) to influence local production of hormones, and regulates satiety and intestinal transit times (129). Propionate and butyrate also mediate anti-inflammatory effects in the host through interaction with GPR43 expressed in Treg cells (in the case of propionate) or interaction with GPR109A on dendritic cells (in the case of butyrate) (129, 130). Of the SCFAs, propionate in particular plays a significant role in modulation of cellular lipid metabolism, resulting in effects that may be linked to the proposed cholesterol-lowering effect of propionate (30). However more studies are required to definitively prove these links and address mechanisms (30). Exposure of rat hepatocytes to propionate in culture resulted in a reduction in cellular cholesterol synthesis (131) an effect that was potentially linked to reductions in acetyl-CoA synthase activity or acetate uptake, both of which are features of cholesterol metabolism [reviewed in Hosseini et al. (30)].

A number of studies have proposed an effect of SCFAs (including propionate) in the lowering of cholesterol markers in animal or human systems. Positive correlations between the cholesterol-lowering properties of probiotics and elevated SCFAs (notably propionate and butyrate) have been made in murine and rat intervention studies (132, 133). Positive correlations have also been made between the cholesterol-lowering properties of fibers other than β-glucan and elevated levels of SCFAs (134, 135).

More direct causal effects can be seen when subjects either consume dietary SCFAs or they are directly infused. In rats dietary supplementation with propionate led to a significant decrease in plasma TC levels (136). A more recent study demonstrated that dietary feeding of individual SCFAs (propionate, acetate or butyrate) was sufficient to lower TC and non-HDL cholesterol in hypercholesterolaemic hamsters (137). The effects were correlated with increased bile acid excretion in the feces and elevated expression of enzymes involved in bile acid synthesis (137). A recent study demonstrated that oral infusion of a mixture of acetate, butyrate and propionate can reduce serum cholesterol levels in pigs (138). In contrast a previous study in which pigs were infused with propionate directly into the caecum failed to show a cholesterol-lowering effect (139). To our knowledge studies investigating the effects of dietary supplementation with SCFAs on cholesterol levels in humans are relatively limited. In two separate studies consumption or infusion of additional dietary propionate did not alter markers of lipid metabolism (140) or cholesterol (141) in healthy volunteers.

### Microbial Exopolysaccharide (EPS) in Cholesterol Homeostasis

In addition to modulation of bile acid profiles and production of SCFAs, gut microorganisms can influence the host through toll-like receptor agonists and other microbial components (including EPS). EPS is composed of repeating carbohydrate moieties, either strongly or loosely associated with the peptidoglycan layer of many lactic acid bacteria (including *Lactobacillus* and *Bifidobacterium* species) (142, 143). Given that these bacterial populations may be altered by consumption of oat β-glucans (19) we predict that EPS is likely to play a role as an effector of microbe-host crosstalk influenced by potential prebiotic effects of β-glucans. EPS is thought to protect the bacterial cell from environmental stressors and to improve survival in the GI tract but also plays a role in microbe-host interactions [reviewed in Ryan et al. (143)]. Production of EPS has been associated with the immunoregulatory properties of specific strains used as probiotics (144) and also plays a role in lowering of cholesterol. The *Pediococcus parvulus* strain 2.6 produces an EPS that resembles the structure of oat β-glucan (143) and the strain has been shown to regulate serum cholesterol in hypercholesterolaemic volunteers consuming a fermented beverage made with *P. parvulus* 2.6 (145). London et al. showed that a *Lactobacillus* strain engineered to produce EPS demonstrated a greater cholesterol-lowering effect in a mouse model of atherosclerosis than an isogenic non-producer (146). Furthermore a *Lb. mucosae* DPC6426 strain which naturally produces high levels of EPS was capable of reducing lipid markers (TC and serum triglyceride) in the same model system (146). EPS extracted from *Lactobacillus* strains caused a reduction in triacylglycerol lipid accumulation in an *in vitro* adipocyte model and a reduction in levels of triacylglycerol and cholesterol in murine fat tissue when mice were injected with EPS. The work demonstrated a role for TLR2 in the cholesterol and lipid-lowering effects of EPS (147). Overall the data suggest that alteration to the relative levels and chemical isotypes of EPS in the GI tract through alterations to the microbiota may have the potential to modulate host cholesterol metabolism, potentially through a TLR2-mediated mechanism. However, further mechanistic studies are required.

### Microbial Cholesterol Assimilation and Metabolism

Numerous bacterial genera found throughout the biosphere have the capacity to metabolize cholesterol. Genomic approaches have identified likely mechanisms by which some species can degrade cholesterol but others remain uncharacterized [reviewed in Garcia et al. (148) and Bergstrand et al. (149)]. A number of gut-dwelling bacterial species have the capacity to transport and/or metabolize cholesterol with the potential mechanisms being established in *Eubacterium coprostanoligenes* (148) an organism that can actively metabolize cholesterol to coprostanol in the GI tract in animal models (150, 151). *Lactobacillus acidophilus, Lb., casei*, and *Lb. bulgaricus* have been shown to assimilate cholesterol and to reduce cholesterol to coprostanol through the activity of a cholesterol reductase (152). Rationally selected *Lactobacillus* strains were capable of reducing serum TC and LDL cholesterol in rats fed a lipid-rich diet, a finding that correlated with elevated SCFAs and bile acid excretion in these animals (132). Recent work has identified that *Bacteroides* spp. isolated from the gut can produce a compound called commendamide which has the capacity to degrade cholesterol and may represent a bacterial adaptation to the gut environment (153). Human intervention studies have indicated an increase in Bacteroidetes in humans following consumption of β-glucan (89), so there is potential for this to represent a mechanism by which microbiota changes may influence cholesterol metabolism in the host. More work is necessary to establish the cholesterol-metabolizing activities of the gut microbiota in health and disease. However, it is clear that alterations to gut microbial community structure have the potential to alter this important physiological function.

## Conclusions and Future Directions

The significant clinical evidence for the cholesterol-lowering effects of β-glucan has led health authorities in the US, Europe and elsewhere to permit health claims attributing a lowering of CVD risk to consumption of specific amounts (generally 3 g per day) of β-glucan. The mechanisms by which β-glucan may lower host cholesterol levels are thought to be linked to an ability to prevent re-circulation or enhance excretion of bile acids, effects that are potentially related to the gel-forming properties of β-glucan. As bile acids are a major repository of cholesterol in the host this leads to an overall reduction in cholesterol from the system.

However, in recent years our knowledge of both cholesterol metabolism and the physiological role of the gut microbiota has increased significantly. It has become clear that diet (including consumption of β-glucans) has the potential to significantly alter the composition of the gut microbiota. In turn studies have shown that the composition of the gut microbiota is a major regulator of both cholesterol and bile acid metabolism in the host. Studies in pigs have shown that β-glucan feeding alters the ability of intestinal cells to reabsorb bile acids and also alters the bile acid profile in the host, suggesting that changes in the microbiota are concomitant with the cholesterol-lowering effect (65). Other studies have confirmed an apparent “prebiotic” effect whereby the microbiota is altered through consumption of oat β-glucan in a manner that is suggestive of an ability to alter the bile acid metabolizing potential of the gut microbial community (19). In the absence of studies which precisely analyze the effect of β-glucan consumption on both the microbiota and bile acid profiles we outlined two hypotheses by which cholesterol metabolism may be impacted by gut microbiota-mediated alterations (section Mechanisms by Which Oat β-Glucan May Influence Host Cholesterol Metabolism Through Alterations in BSH Activity of the Microbiome). We propose a microbe-centered model in which microbial bile acid metabolism results in reduced engagement of the host bile acid receptor FXR, stimulating enhanced *de novo* bile acid synthesis and enhanced TICE (Figure 1). Furthermore, in this review we outline that other microbe-host interactions may contribute to the cholesterol-lowering effects of β-glucan though stimulation of SCFA production, cholesterol degradation or via the effects of microbial EPS.

We propose that future studies should utilize a systems biology approach toward understanding the complex interplay between β-glucan, the microbiota and mechanisms in the host that regulate serum cholesterol levels. Data which links consumption of β-glucan to bile acid changes in the host and identifies host metabolic changes (including to levels of FGF19) will be invaluable for enhancing our understanding of the mechanisms by which oat β-glucan mediates its cholesterol-lowering effects.

## Author Contributions

SJ and CG wrote and edited the manuscript. AK and LF edited, provided critical feedback, and contributed significantly to the writing of the manuscript.

### Conflict of Interest

AK and LF are employed by PepsiCo, Inc. The remaining authors declare that the research was conducted in the absence of any commercial or financial relationships that could be construed as a potential conflict of interest.
